# First report of *Rickettsia felis* in mosquitoes, USA

**DOI:** 10.1080/22221751.2020.1760736

**Published:** 2020-05-18

**Authors:** Subarna Barua, Md Monirul Hoque, Patrick John Kelly, Anil Poudel, Folasade Adekanmbi, Anwar Kalalah, Yi Yang, Chengming Wang

**Affiliations:** a Department of Pathobiology, Auburn University College of Veterinary Medicine, Auburn, AL, USA; c Department of Clinical Sciences, Ross University School of Veterinary Medicine, Basseterre, Saint Kitts and Nevis; g College of Veterinary Medicine, Yangzhou University, Yangzhou, People’s Republic of China; h Department of Pathobiology, Auburn University College of Veterinary Medicine, Auburn, AL 36832, USA.

The recently described *Rickettsia felis* is an emerging human pathogen causing flea-borne spotted fever [1]. Although found in a wide range of arthropods including fleas, ticks, mites, and lice, the cat flea (*Ctenocephalides felis*) is currently believed to be the most likely primary vector of *R. felis*. There is growing evidence, however, that mosquitoes might be involved in *R. felis* transmission with the organism having been identified in a wide variety of mosquitoes in Africa and China [1,2]; mouse-model experiments have indicated transmission of *R. felis* by *Anopheles gambiae* [3]; there is an association between malaria and flea-borne spotted fever cases in Africa [2]. Although *R. felis* has been demonstrated in a variety of mammals and arthropods in the USA, there is only one study on its presence in mosquitoes [4]. The organism was not identified by PCR in pools of *Culex quinquefasciatus, Aedes albopictus, Culex pipiens* complex, *Anopheles punctipennis* and *Anopheles crucians* from Georgia [4].

To further investigate *R. felis* in mosquitoes in the USA, we studied 560 unfed adult mosquitoes trapped with CDC miniature light traps (John W. Hock, Gainesville, FL) throughout October 2019 on the campus of Auburn University College of Veterinary Medicine, Alabama. The mosquitoes were identified morphologically and with a PCR targeting the mitochondrial cytochrome c oxidase subunit [5] before being pooled (n = 57; 4 to14 per pool) according to species, sex, trap number and collection site. After washing (once in PBS for one minute; once in 70% ethanol for ten minutes; four times, one minute each, in sterile PBS) to remove surface contaminants, DNA was extracted for three previously published and validated PCRs, a *gltA*-based FRET-PCR [3], a nested-PCR targeting the *gltA* of *Rickettsia* [3], and a *R. felis* species-specific *BioB*-based PCR [4], which were performed to test for the presence of *Rickettsia* DNA in mosquitoes.

**Figure 1.**
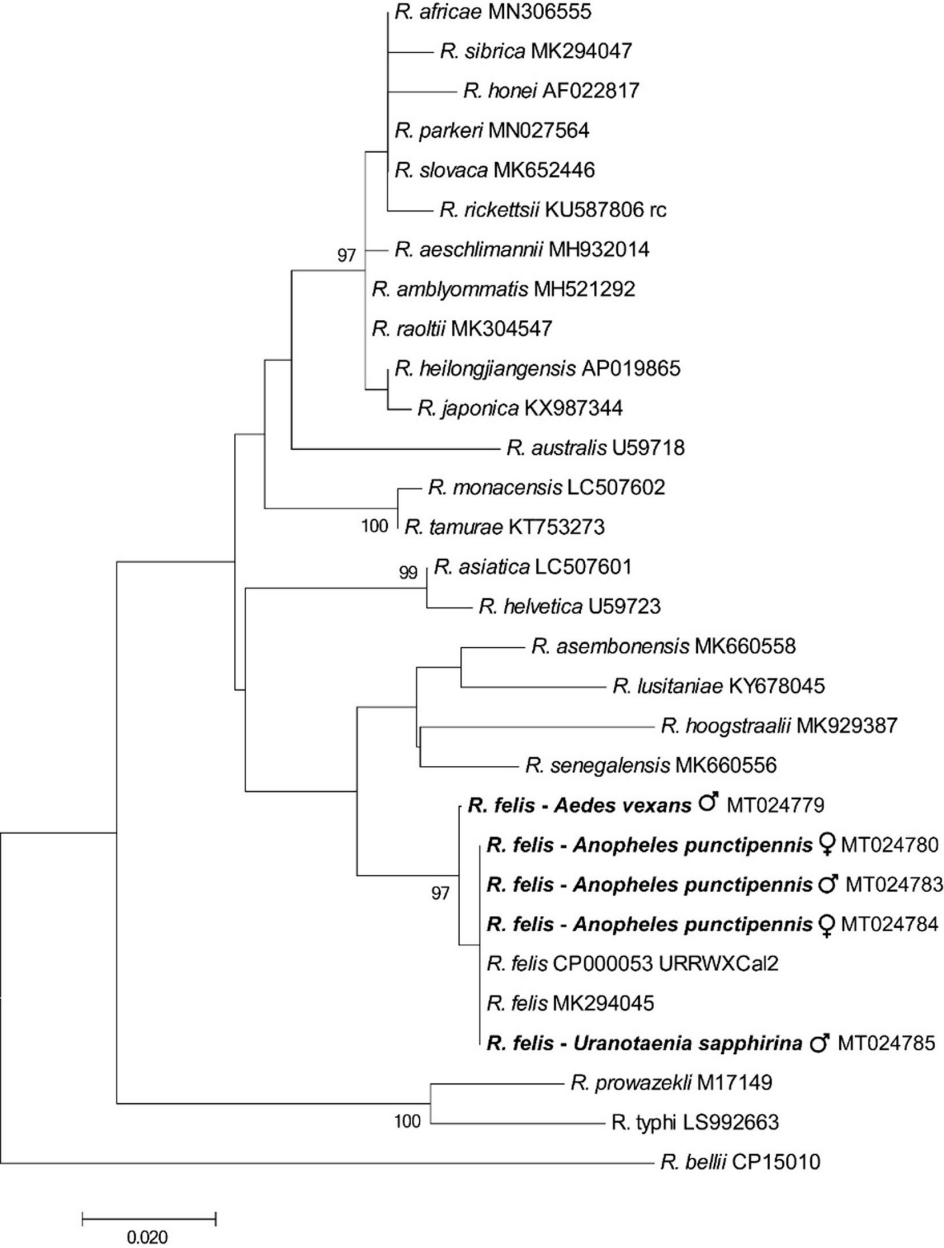
Phylogenetic tree using a bootstrap analysis for the *Rickettsia felis* found in mosquitoes from USA. The 446-bp nucleotide sequences of the *gltA* PCR products were concatenated and aligned using CLUSTALW, and the phylogenetic inferences were obtained from a maximum likelihood analysis. The names of *Rickettsia* species and their GenBank accession numbers are provided. The numbers at the nodes are the bootstrap values obtained by repeating the analysis 100 times to generate a majority consensus tree. The sequences of *R. felis* identified in this study (in bold) were 99.7–100% identical to the recognized *R. felis* strains, and 84.0–95.7% identical to other *Rickettsia* spp. The bootstrap values < 80 were omitted in the phylogenetic tree.

Nine percent (5/57) of the mosquito pools, including *An. punctipennis* (3/6), *Aedes vexans* (1/4) and *Uranotaenia sapphirina* (1/3), were positive by PCR, in each case with all three PCRs. One of the positive *An. punctipennis*, one of the positive *Aedes vexans* and the positive *U. sapphirina* pool contained only male mosquitoes. The remaining pools were negative: *Culex erraticus* (5), *Culex nigripalpus* (8), *Culex coronator* (2), *Culex conspirator* (9), *Culex tarsalis* (1), *Cx. pipiens* (13), *Culex territans* (1), *Culex restuans* (2), *Cx. quinquefasciatus* (2) and *Ae. albopictus* (1). The 120-bp nucleotide sequences of the five mosquito pools positive in *R. felis*-specific *BioB*-based PCRs were identical to one another, and to that of *R. felis* URRWXCal2 (CP000053.1). There was only a single base pair difference amongst the 446-bp nucleotide sequences of the positive *gltA*-based PCRs which were 99.7–100% identical to recognized *R. felis* strains in GenBank, and 84.0–95.7% identical to other *Rickettsia* spp. (Figure 1). Most closely related organisms were *Rickettsia lusitaniae, Rickettsia hoogstraalii* and *Candidatus Rickettsia senegalensis* with which *R. felis* is known to cluster [1].

Our results show *R. felis* occurs in mosquitoes in the USA and adds *An. punctipennis*, *Ae. vexans* and *U. sapphirina* to the mosquitoes known to harbour the organism. Further more extensive studies are needed to determine the range of mosquitoes harbouring *R. felis* across the USA. It is noteworthy that one positive pool of *An. punctipennis*, one positive pool of *Ae. vexans* and one positive pool of *U. sapphirina* contained only male mosquitoes. As males do not take blood meals, it appears likely the infections were congenital and vertical transmission occurs in these mosquito species.

Opossums are the probable main mammalian reservoirs of *R. felis* in endemic areas in the USA [4] while dogs have been implicated elsewhere in the world [6]. None of the mosquitoes we found positive for *R. felis* have a reported tendency to feed on these species. *An. punctipennis* occurs widely in the eastern USA and feeds mostly on mammals, especially deer and sheep, but also on birds and people [7]. The cosmopolitan *Ae. vexans* feeds on people and other mammals, especially deer, cattle, horses, rabbits, sheep, and dogs [8]. *U. sapphirina* is found in the eastern, central and southern US, and is the only mosquito known to feed on invertebrates, mainly earthworms and leeches [9]. Our finding that this species was infected with *R. felis* is of note as leeches have previously been suggested to be vectors of the organism [10].

There is still much to be understood about the vector and reservoir role of the wide range of arthropods that harbour *R. felis.* The growing reports of *R. felis* occurring in mosquito species around the world and the known role of mosquitoes in transmitting a wide range of very important human and animal pathogens indicate an urgent need for further studies to determine the role mosquitoes might play in the epidemiology of *R. felis* infections in people. Further, since *R. felis* might play a role in parthenogenesis in arthropods [1], its role in the biology of mosquitoes would thus also appear to warrant detailed investigation.
